# Access to *PAIN Reports* is now open: IASP inaugurates a new journal

**DOI:** 10.1097/PR9.0000000000000563

**Published:** 2016-08-09

**Authors:** David Yarnitsky, Francis J. Keefe

**Affiliations:** a Department of Neurology, Rambam Medical Center, Haifa, Israel,; b Pain Prevention and Treatment Research, Department of Psychiatry and Behavioral Sciences, Duke University, Durham, NC, USA

We are excited to announce the launch of an open access journal from IASP called *PAIN* *Reports*. The new online journal joins *PAIN* in offering IASP members and the pain research community 2 publishing venues for the best basic and applied pain research. Although *PAIN* will continue to publish the foremost research articles in the field, *PAIN Reports* will offer readers worldwide free access to scientific articles and will expand the publishing terrain for developing countries.

Why has IASP entered the field of open access publishing? In an era of easy and common internet access and ubiquitous communications technology, the scientific landscape continues to change before our eyes. Compared with just a few years ago, all of us communicate far more often, more rapidly, and more intensively with our peers around the globe. Communication, of course, is at the heart of our scientific enterprise; scientists and publishers, therefore, cannot refrain from adjusting to this new wave.

Today, online scientific journals are proliferating in various fields, even as freedom of access to data remains somewhat elusive. Yet momentum clearly is on the side of open access publishing. The rationale is simple: taxpayer money is being used, justifiably, to finance research, but the results of this research generally are unavailable to the public. In most cases, only members of the scientific community have easy access to journal articles.

The open access vision, accordingly, is that all data will be freely accessible to all stakeholders—the public, health policy experts, and the broader scientific community. Money allocated for research increasingly will include funds for open access publication as part of the expense of research.

Several steps in this direction already are evident. PubMed Central, acting under the US Consolidated Appropriations Act, 2008, publishes full-text versions of government-funded research, making these articles publicly accessible. Similar steps have been taken in the United Kingdom, with UK PubMed Central, as well as in Europe and Canada. The Dutch government, currently pursuing a pan-European open science policy platform, aims to shift scientific publications toward open access venues.^2^

Many medical societies now have at least 1 open access journal, and nearly all scientific publishers support hybrid options, in which authors pay fees to publishers in return for their articles to be open access. (*PAIN* has this option for authors; as an open access journal, *PAIN Reports* charges authors publishing fees). For example, the Public Library of Science (PLOS) publishes a major line of open access journals. The most popular of these, *PLoS One* is one of the largest scientific journals, with approximately 30,000 articles published annually. Overall, there is evidence that the percentage of immediate open access articles has increased from 4.6% of all scientific articles in 2006 to 11% in 2011. If articles that are opened after a 1-year embargo and those published in hybrid journals are added, the numbers increase to 8.1% and 16.9%, respectively.^1,3^

As an online-only journal, *PAIN Reports* hopes to publish a high volume of articles rapidly and substantially complement *PAIN* in generating a multifaceted platform for clinical and research communications in pain research and management.

The mission statement of *PAIN Reports* asserts our commitment:

*PAIN Reports* is an official IASP publication. An open access multidisciplinary journal, *PAIN Reports* promotes a global, rapid, and readily accessible forum that advances clinical, applied, and basic research on pain. The online journal publishes full-length articles as well as brief reports, reviews, meta-analyses, meeting proceedings, and selected case reports. *PAIN Reports* gives special attention to submissions reporting the results of enterprising and high-risk research and pilot studies as well as locally developed clinical guidelines from scientists and clinicians in developing countries.

It is with great excitement that we call for submissions from pain professionals in the clinical and research communities to support *PAIN Reports*.

## Conflict of interest statement

The authors have no conflicts of interest to declare.
